# Association between novel TARDBP mutations and Chinese patients with amyotrophic lateral sclerosis

**DOI:** 10.1186/1471-2350-11-8

**Published:** 2010-01-19

**Authors:** Hui-Ling Xiong, Jin-Yang Wang, Yi-Min Sun, Jian-Jun Wu, Yan Chen, Kai Qiao, Qiao-Juan Zheng, Gui-xian Zhao, Zhi-Ying Wu

**Affiliations:** 1 Department of Neurology and Institute of Neurology, Huashan Hospital, State Key Laboratory of Medical Neurobiology, Shanghai Medical College, Fudan University, 12 Wulumuqi Zhong Road, Shanghai 200040, PR China; 2 Department of Neurology and Institute of Neurology, First Affiliated Hospital, Fujian Medical University, 20 Chazhong Road, Fuzhou 350005, PR China

## Abstract

**Background:**

*TARDBP *mutations have been reported in patients with amyotrophic lateral sclerosis (ALS) in different populations except Chinese. The present aim is to investigate the association between *TARDBP *mutations and Chinese patients with ALS.

**Methods:**

71 SALS patients and 5 FALS families with non-*SOD1 *mutations were screened for *TARDBP *mutations via direct sequencing.

**Results:**

A novel heterozygous variation, Ser292Asn (875G>A), was identified in the proband and 4 asymptomatic relatives including the children of the dead patient from a FALS family. Thus the dead patient, the proband's brother, was speculated to carry Ser292Asn though his sample was unavailable to the detection. This variation was not found in 200 unrelated control subjects. A homology search of the TDP-43 protein in different species demonstrated that it was highly conserved. Also, it was predicted to be deleterious to protein function with SIFT-calculated probabilities of 0.00. Therefore, Ser292Asn is predicted to be a pathogenic mutation. In addition, we have found two silent mutations (Gly40Gly and Ala366Ala) and one novel polymorphism (239-18t>c).

**Conclusions:**

The present data have extended the spectrum of *TARDBP *mutations and polymorphisms, and supported the pathological role of TDP-43 in Chinese ALS patients.

## Background

Amyotrophic lateral sclerosis (ALS) is the most common form of motor neuron disease and is characterized by progressive loss of upper and lower motor neurons from the spinal cord, brain stem and cerebral cortex, leading to paralysis and eventually death which is due to respiratory failure within 3-5 years after symptom onset [1]. Approximately 5-10% of ALS cases are familial (FALS) whereas the remaining patients are sporadic (SALS) [2]. About 15-20% of patients with autosomal dominant FALS have mutations in the copper-zinc superoxide dismutase 1 gene (*SOD1*), while mutations in other genes including alsin (ALS2), senataxin (*SETX*, ALS4), dynactin (*DCTIN1*), angiogenin (*ANG*), synaptobrevin-associated membrane protein B (*VAPB*, ALS8) and Fused in Sarcoma (*FUS*, ALS6) are described as rare causes of FALS [2-5]. Recently, a novel senataxin mutation has been reported in a SALS patient [6]. Ubiquitin-positive tau-negative neuronal cytoplasmic inclusion is the common pathological feature in frontotemporal lobar degeneration (FTLD) and ALS. TAR DNA-binding protein 43 (TDP-43), which is a 414-amino-amino-acid nuclear protein encoded by *TARDBP *on chromosome 1p36.22, was recognized as a major constituent of these neuronal cytoplasmic inclusions [7,8]. To date, a total of 30 *TARDBP *mutations have been reported in ALS patients [9-20]. These mutations affect the C-terminal region of TDP-43 and may influence protein-protein interaction, exon skipping and splicing inhibitory activity, thus, may influence the proper function or transport of TDP-43. Here, we first reported the screen for *TARDBP *mutations in Chinese patients with ALS and we identified one novel missense mutation, two silent mutations and one novel polymorphism.

## Methods

### Subjects

Seventy-one unrelated SALS patients and probands from 5 FALS families with non-*SOD1 *mutations from the Han ethnic group were enrolled in our study between 12 December 2007 and 3 March 2009 from department of Neurology at Huashan Hospital. Of them, 56 were males and 20 were females, and the average age of symptom onset was 52 years (range, 32-76 years). All patients had been examined by at least two neurologists. Neurological examinations including electromyography (EMG) and magnetic resonance imaging (MRI) of the cervical cord were performed. Medical history and demographic information were collected by a specially-assigned person, and records were reviewed by two senior neurologists. All patients were diagnosed as definite ALS according to the Airlie House criteria [21]. 16 patients presented with bulbar-onset disease and 60 patients presented with spinal-onset disease. Two hundred unrelated aged individuals (≥60 years) consisted of 100 men and 100 women with no known history of ALS were selected as a control group. All of them are Han people from Southern China. This study was approved by the local ethics committee, and informed consent was obtained from the participants or their legal surrogates prior to inclusion in the study. Genomic DNA was extracted from peripheral EDTA blood with a TIANamp Blood DNA kit (TIANGEN Biotech, Beijing).

### Mutation scanning

The coding region of *TARDBP*, exons 2-6, including the intron-exon boundaries, were analyzed using primer combinations designed based on the intronic sequences of *TARDBP*. PCR amplification was performed using a GeneAmp PCR system 9700 (Applied Biosystems, Foster City, CA, USA) with standard conditions. The sequence of the primers and the annealing temperatures are shown in the Table 1. Amplified products were purified and subjected to direct sequencing, and the procedure is as previously reported [22]. Obtained sequences were compared with the genomic DNA sequence of *TARDBP *(NCBI Sequence Viewer NT_021937.18), and nucleotide changes were numbered corresponding to their position in *TARDBP *mRNA (NCBI Sequence Viewer NM_007375.3).

**Table 1:** Primers designed for the *TARDBP *gene and conditions of PCR

Primers	Oligonucleotide of primers	Size of PCR product	Annealing temperature
Exon2	F 5'-CTGGAAGTCAGAACTCTGAC-3' R 5'-TCAGGAGACATTCTGCCACC-3'	447 bp	66°C
Exon3	F 5'-GCTTCTCATTTCTAGATGTAGG-3' R 5'-AGAACCTAGGGAACATAGTG-3'	357 bp	58°C
Exon4	F 5'-TAAGCCACTGCATCCAGTTG-3' R 5'-GATTTCATGAACACACCCTG-3'	367 bp	66°C
Exon5	F 5'-TGGTTCACTGCTATCCAAGG-3' R 5'-AGGATGGTCTTGATCTGGTG-3'	396 bp	60°C
Exon6	F 5'-CATTGCTTATTTTTCCTCTGGC-3' R 5'-TATACTCCACACTGAACAAACC-3'	780 bp	62°C

## Results

### Mutations and polymorphisms of TARDBP gene identified in the present study

**Figure 1:**
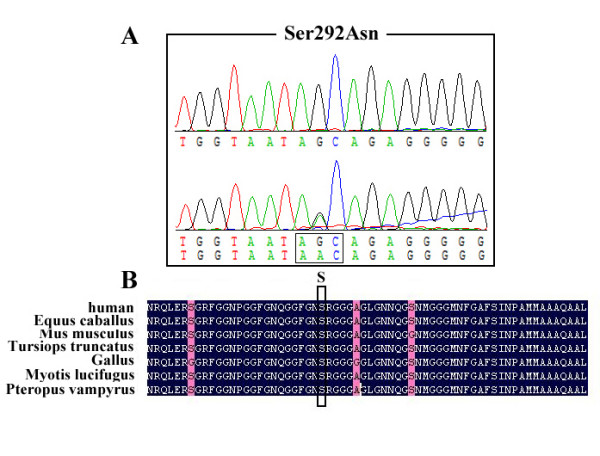
**Chromatogram of Ser292Asn (A) and conservation of Ser292Asn in different species (B)**.

After screening mutations of *TARDBP *in 5 FALS patients and 71 SALS patients, a heterozygous variation Ser292Asn (875G>A) which has not been reported previously was identified in a FALS patient. The chromatogram is shown in figure 1A. This variation was not found in 200 unrelated control subjects, reducing the likelihood that it represents a polymorphism. Moreover, a homology search of the TDP-43 protein in different species demonstrated that this variation was highly conserved (figure 1B). At the same time, the program SIFT (Sorting Intolerant From Tolerant) [23] has been applied to predict whether this sequence change could affect protein function, and it was predicted to be deleterious to protein function with SIFT-calculated probabilities of 0.00, thus Ser292Asn is a novel mutation and most probably a pathogenic one.

In addition, two silent mutations, Gly40Gly (120G>A) and Ala366Ala (1098C>G) were detected. The Gly40Gly which has not been reported previously was found in one SALS patient and none of 200 unrelated control individuals, indicating that it is a rare silent mutation. The Ala366Ala which has been previously reported in Japanese SALS patients [20] was found in one SALS patient and one control individual, thus it was thought to be a benign polymorphism. We also have detected a novel polymorphism 239-18t>c in one SALS patient and one control individual. The chromatograms of them are shown in additional file 1.

#### Clinical features of the FALS patient carrying Ser292Asn mutation

The Ser292Asn mutation was identified in a 67-year-old male FALS patient (II_2_). He had some difficulty in speech at the age of 64 and gradually presented dysarthria, dysphagia and atrophy of lingual muscle. One year later, he began to have some difficulty in using chopsticks and lifting his right arm, and gradually developed weakness of both upper limbs and atrophy of muscles. However, the fasciculation was slight. After another 4 months, he began to feel weakness in both lower extremities and started having difficulties in walking up and down stairs. Neurological examination showed muscle weakness involving all the extremities with motor power graded as follows: upper limbs, 3/4; lower limbs, 4/5. However, the sensation was intact. Atrophy of lingual muscle and distal muscles of upper limbs were evident. There was positive Babinski sign, and the deep-tendon reflexes were increased in both lower limbs whereas decreased in both upper limbs. EMG testing showed reduced amplitudes of compound muscle-action potentials in right median nerve, positive sharp waves, fibrillations, and normal motor and sensory NCV. He was treated with coenzyme Q10 60 mg daily and followed with follow-up examinations every three months. At the last follow-up 2 months ago, neurological examination showed progression of disease. The powers of upper limbs were both 2/3 grades and powers of lower limbs were both 3/4 grades. Dysarthria and dysphagia were more and more evident, whereas the patient refused Bi-level positive airway pressure (BiPAP) and percutaneous endoscopic gastrostomy (PEG).

#### Pedigree analysis of the ALS family with Ser292Asn mutation

**Figure 2:**
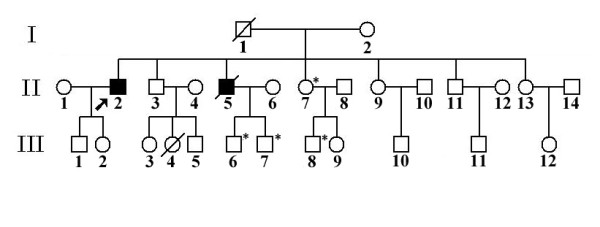
**The pedigree with Ser292Asn**. "*" indicates the asymptomatic relatives carrying Ser292Asn.

The pedigree of this family is shown in figure 2. The proband's younger brother (II_5_) developed right lower limb weakness at the age of 58, and gradually extended to muscles of both lower limbs and upper limbs. Two years later, he developed bulbar symptoms and died of respiratory failure at the age of 61.

Direct sequencing was performed to screen for Ser292Asn in the proband's mother (I_2_), three sisters (II_7_, II_9 _and II_13_), one brother (II_11_), and their children (III_3_, III_5_, III_6_, III_7_, III_8_, III_11_). Four of them (II_7_, III_6_, III_7_, III_8_) were detected to have Ser292Asn. They haven't any clinical symptoms presently. II_7 _is 60 years old while III_6_, III_7 _and III_8 _are less than 40 years old. Because the 85-year-old mother (I_2_) doesn't have Ser292Asn, we speculated that Ser292Asn might be come from the dead father who was asymptomatic. III_6 _and III_7 _are the children of the dead patient (II_5_), thus we speculate that II_5 _carried the Ser292Asn mutation, although his sample is unavailable for confirmation.

## Discussion

The TDP-43, which is a 414-amino-amino-acid nuclear protein encoded by *TARDBP *on chromosome 1p36.22, has been identified as the major disease protein in ALS. It is evolutionary conserved and its structure consists of a glycine-rich domain and two RNA recognition motifs [24]. It is known to bind DNA and RNA, such as human immunodeficiency virus type 1 TAR DNA sequence motifs [25], and to be involved in the regulation of messenger RNA splicing and exon skipping [26]. The importance of the glycine-rich C-terminal domain of TDP-43 in mediating its exon skipping and splicing inhibitory ability has been demonstrated and has been observed to correlate with its ability to interact with other members of the heterogeneous nuclear ribonuclear A and B protein families with well-known splicing inhibitory properties [27]. However, the exact function remains unclear.

Recently different pathogenic *TARDBP *mutations have been described in different cohorts of patients with FALS and SALS, supporting a direct role of TDP-43 in neurodegeneration [9-20]. However, two additional studies by Gijselinck I et al and Guerreiro RJ et al failed to find mutations in Belgian and North American patients with ALS [28,29]. Among the 30 previously reported mutations [9-20], 29 are missense mutations, and one is frameshift mutation (Tyr374Term) which creates a premature stop codon, consequently leading to the expression of a truncated protein [17]. None of them is homozygous. Met337Val, Gly348Cys and Ala382Thr are the most common mutations. Additionally, except for the Asp169Gly mutation, all other *TARDBP *mutations are located in exon 6 encoding for the C-terminus of TDP-43. Considering all the previous studies, the frequency of *TARDBP *mutations is 3.6% in FALS and 1.0% in SALS [16]. Most ALS patients carrying *TARDBP *mutations have an Italian or French origin, suggesting a higher frequency of *TARDBP *mutations in Southern Europe (average 3.4%; 8% in France and 2.7% in Italy) than in other Caucasian populations (average 0.7%) [16].

In the present study, after screening *TARDBP *mutations in 71 unrelated SALS patients and probands from 5 FALS families in Chinese population, we have identified one missense mutation in a FALS family and 2 silent mutations in SALS patients. This frequency is lower than those of previous studies, which may be due to the cohort of ALS patients analyzed here is small compared to previous studies. Also, different races may refer to different genetic backgrounds. The Ser292Asn mutation was located in the highly conserved region, exon6, encoding for the C-terminus of TDP-43. In the FALS family with Ser292Asn, we speculated that I_1 _and II_5 _might both carry Ser292Asn though their samples are unavailable to the detection. The proband presented clinical symptoms at the age of 64. However, the other four family members (II_7_, III_6_, III_7_, III_8_) carried the same mutation as the proband did were asymptomatic presently. III_6_, III_7 _and III_8 _are in their thirties and obviously haven't reached the onset age of ALS. II_7 _is 60 years old though she is clinically asymptomatic. We suggest detecting EMG examination to her but she refused.

## Conclusion

In conclusion, our data have extended the spectrum of *TARDBP *mutations and polymorphisms, and supported the pathological role of TDP-43 in ALS. Further studies are needed to shed light on the pathophysiological link between Ser292Asn and ALS. Competing interestsThe authors declare that they have no competing interests.

## Authors' contributions

**Hui-Ling Xiong **carried out the molecular genetic studies, participated in the analysis of the data and drafted the manuscript. **Jin-Yang Wang **and **Yi-Min Sun **collected demographic data and risk factor profiles of subjects and participated in analysis and interpretation of data. **Jian-Jun Wu, Yan Chen **and **Kai Qiao **analyzed the clinical data of all subjects. **Gui-xian Zhao **and **Qiao-Juan Zheng **participated in the acquisition of data. **Zhi-Ying Wu **conceived of the study, and participated in its design and coordination, and revising the manuscript critically for important intellectual content. All authors have read and approved the final manuscript.

## Pre-publication history

The pre-publication history for this paper can be accessed here:

http://www.biomedcentral.com/1471-2350/11/8/prepub

## Supplementary Material

Additional file 1 **Chromatograms of silent mutations (Gly40Gly and Ala366Ala) and the novel polymorphism (239-18t>c). **The normal sequence is shown in the upper half and the corresponding mutation is shown below. Click here for file
