# Supplementary figures and images for: Association between novel TARDBP mutations and Chinese patients with amyotrophic lateral sclerosis
Source: BMC Med Genet. 2010 Jan 19;11:8. doi: 10.1186/1471-2350-11-8 (PMC2821387; doi:10.1186/1471-2350-11-8)

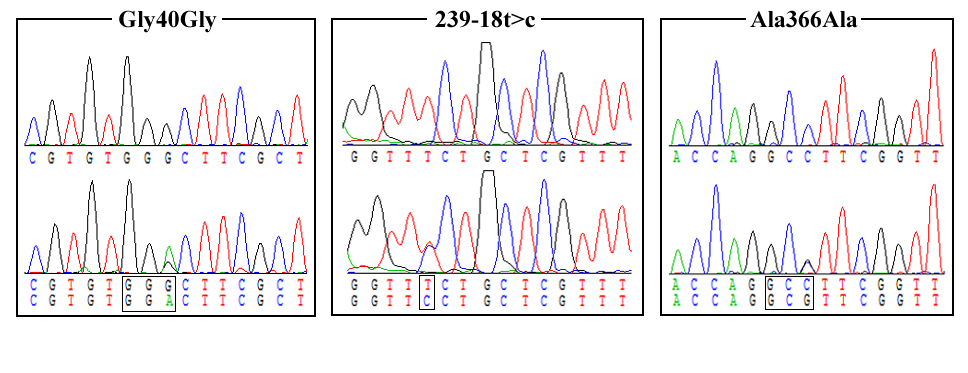

Supplement: Additional file 1 — Chromatograms of silent mutations (Gly40Gly and Ala366Ala) and the novel polymorphism (239-18t>c). The normal sequence is shown in the upper half and the corresponding mutation is shown below. [file 1471-2350-11-8-S1.JPEG]
